# How global spine sagittal alignment and spinal degeneration affect locomotive syndrome risk in the elderly

**DOI:** 10.1007/s11845-024-03813-3

**Published:** 2024-10-01

**Authors:** Ramada R. Khasawneh, Ejlal Abu-El-Rub, Rawan. A. Almazari, Ayman G. Mustafa

**Affiliations:** 1 Department of Basic Medical Sciences, Faculty of Medicine, Yarmouk University, Irbid, Jordan; 2 Department of Basic Medical Sciences, College of Medicine, QU Health, Qatar University, Doha, Qatar

**Keywords:** Elderly, Locomotive syndrome, Lumbar disc height, Middle east, Spine sagittal alignment

## Abstract

**Background:**

The aim of this study was to delineate the features of the locomotive syndrome (LS) risk stage in the elderly population, encompassing global spine sagittal alignment, visible spinal degenerative changes on plain radiographs, muscle strength, physical capabilities, and low back pain (LBP).

**Methods:**

The study enrolled 232 subjects, evaluated using plain radiographs. The evaluation included measurements of lumbar lordosis (LLA), thoracic kyphosis (TKA), spinal inclination (SIA) angles, and sagittal vertical axis. Assessments included lumbar osteophyte formation (LOF) and lumbar disc height (LDH) to examine spinal degenerative changes. LS evaluation used the locomotive syndrome risk test based on LS risk criteria, classifying participants into no risk, stage 1 LS, and stage 2 LS groups. Using a visual analogue scale (VAS), we investigated the prevalence of low back pain (LBP) and assessed physical performances across these groups.

**Results:**

There were 132 participants with no LS risk, 71 with stage 1 LS risk, and 29 with stage 2 LS risk. As LS risk increased, LBP prevalence and VAS scores rose, physical abilities, and back muscle strength decreased. TKA showed no variation across groups, while LLA decreased with advancing LS risk stage. Except for L1–L2 and L5–S1, lumbar disc height (LDH) decreased with higher LS risk stages. LOF occurrence increased notably with higher LS risk stages. Spinal inclination angle (SIA) significantly increased with advancing LS risk stages.

**Conclusion:**

Participants diagnosed with LS exhibited an increased incidence of spinal degeneration, reduced LLA, and global spinal imbalance characterized by anterior spinal inclination.

## Introduction

The locomotive system, also known as the musculoskeletal system, is a complex network of bones, muscles, joints, tendons, and ligaments that enables movement and provides structural support to the human body. This system is essential for various functions, including posture maintenance, locomotion, and the performance of daily activities [1]. Locomotive syndrome (LS) is a medical condition characterized by a decline in mobility due to dysfunction in the musculoskeletal system [1]. Primarily affecting older adults, LS results in difficulties with walking, maintaining balance, and performing daily activities [1]. As the global population ages, the prevalence of LS is increasing, making it a significant public health concern. The condition not only impacts physical health but also affects psychological well-being and social participation, underscoring the need for comprehensive approaches to prevention, diagnosis, and management [2].

According to the global classification system, LS is categorized into three stages, each representing different levels of severity and risk for functional decline [3, 4]. Understanding these stages is crucial for early detection, effective intervention, and prevention of further disability. This comprehensive look into the characteristics of each stage provides insights into how LS develops and progresses. Stage 1 of LS is characterized by mild symptoms that indicate the initial decline in physical function [3]. Individuals in this stage may experience occasional joint stiffness, minor pain during movement, and slight difficulty in performing physically demanding tasks [3].

Stage 2 of LS marks a more noticeable decline in mobility and physical function. Symptoms become more pronounced, with individuals experiencing increased pain, reduced range of motion, and greater difficulty in maintaining balance [3]. Daily activities such as climbing stairs, walking long distances, and carrying heavy objects become challenging and may require assistance. The risk of falls and related injuries is significantly higher in this stage due to the compromised musculoskeletal system [3]. Finally, stage 3 represents the most advanced stage of locomotive syndrome, where individuals face severe limitations in their ability to move and perform daily activities independently [3].

Over the past two decades, the Middle East has experienced a significant demographic shift characterized by a rapidly ageing population [5]. One of the most pressing health issues among the elderly is the rise in musculoskeletal disorders and the LS, which significantly impact mobility and quality of life such as development of spinal kyphosis, chronic pain, poor muscle strength, and poor physical ability. The literature highlights a significant correlation between spinal sagittal alignment and quality of life, with a specific focus on the Japanese population [6–9]. However, none of the studies examined the effects of spinal sagittal alignment and spinal degradation at various stages of LS risk.

In this study, we unravel the possible relationship between spinal sagittal alignment and spinal degeneration across various stages of LS within a healthy Arabic population. Our research aimed to shed light on these dynamics to better establish of effective preventive strategies and treatment approaches.

Additionally, this study is groundbreaking in the Arab world and Middle East as it delineates the features of each LS risk stage, encompassing global spine sagittal alignment, visible spinal degenerative changes on plain radiographs, and aspects such as low back pain (LBP), muscle strength, and physical capability among elderly population.

## Methods

### Study population

Approval for this study was granted by the institutional research board at Jordan University of Science and Technology (IRB #7/134/2022). Written informed consent was collected from all participants.

Participants were recruited from King Abdullah University Hospital in Irbid, Jordan, where they had participated in a basic health checkup organized by both the hospital and university in 2022. Those with a history of lower back pain, vertebral column fractures, dislocations, spinal cord injuries, or knee surgeries were excluded from the study; moreover, this study did not include any participants with stage 3 LS. A visual analogue scale (0 to 100 mm, VAS) was utilized to investigate the prevalence of LBP and sciatica [10] to excluded them as well.

The study included 232 subjects (101 men and 131 women; mean age, 63.9 years) who underwent physical performance tests, the LS risk test, and whole spine radiography. Body weight (kg) and height (cm) were recorded, and body mass index (BMI) was computed. Participants with a BMI exceeding 30 were excluded to minimize the impact of obesity-related confounding factors.

### Physical performance tests

Using a digital meter, back muscle strength was assessed by measuring maximal isometric strength of trunk muscles in a standing position with 30° lumbar flexion [11]. The evaluation of all participants was performed by a single examiner who remained unaware of the results from other evaluations.

For the physical performance, the participants were requested to walk a straight 10 m at their maximum speed [11, 12].

Subsequently, participants undertook the 3-m timed up-and-go test (3-m TUG), which involved standing up from a standard armchair, walking 3 m to a marker, turning, walking back, and sitting down. The time taken to complete this sequence of actions is recorded.

Finally, participants did the maximum stride length test, the participants are instructed to take the longest step possible forward with one leg while maintaining their balance and without overstepping, this sequence was performed twice, once with the right foot leading and then with the left foot leading [13].

### Locomotive syndrome risk test

This test consisted of three parts: the two-step test, stand-up test, and the 25-question Geriatric Locomotive Function Scale (GLFS-25) [14, 15].

For the two-step test, we marked a starting line on the floor, then asked the participant to stand with their toes on this line and then take two consecutive steps as far as possible, trying to maximize the distance covered [14, 15]. The two-step test score was calculated by dividing the combined length of the two steps (in centimeters) by the individual’s height (in centimeters).

For stand-up test, we asked the participant to sit in the chair and then stand up without using their hands for support. This test addresses the difficulties in one-leg rising from a 40-cm-high seat, with difficulty or a failure indicating stage 1 LS risk, and rising from a 20-cm-high seat with both legs, where difficulty or a failure indicating stage 2 LS risk [14, 15].

Finally, we asked the participants to fill a self-administered questionnaire designed to capture a comprehensive picture of the participant’s locomotive function, which includes 25 questions covering aspects such as pain, daily activity limitations, and social interactions. Items are scored on a 0 to 4 scale, with higher values showing greater difficulty or impairment. The overall score is obtained by summing the individual responses, ranging between 0 and 100, with higher totals reflecting more severe LS impairment [16, 17].

### X-ray imaging assessment

All images were obtained from Siemens digital radiography systems (Siemens Healthineers, Germany). The participants were standing with hands on clavicles so as to avoid obscuration or over lapping shadows. The average settings were 90 kV and 100 mAs for the lateral X-ray. The lateral X-ray images were obtained and evaluated with measurement software. Three angles were measured using the lateral view: (1) the angle between superior endplates of L1 to S1 or the lumber lordosis (LL angle), (2) thoracic kyphosis (T4–L1, TK), and (3) the sagittal vertical axis (C7–S1, SVA).

To evaluate spinal degenerative changes, lumbar disc height (LDH) and lumbar osteophyte formation (LOF) were studied across the levels from L1-L2 to L5-S1 as reported [18]. LOF at each level of spinal segment was categorized using the Nathan classification (0–4) [19]. The incidence of LOF was assessed and contrasted across three subject groups: those with no risk, stage 1 LS risk, and stage 2 LS risk categories. Two expert spinal surgeons conducted the measurement of each parameter using Surgimap (Surgimap Spine Software, Nemaris Inc., New York, NY, USA).

### Data analysis

Nonparametric data differences between groups were evaluated using the Mann–Whitney *U* test, and three-group comparisons were done using the Kruskal-Wallis test followed by the Mann–Whitney *U* test. Correlations were determined by Spearman’s rank correlation coefficient, and the chi-square test analyzed group differences. Statistical significance was set at a *P* value of less than 0.05. Data were expressed as mean ± standard error of the mean (SEM).

## Results

Out of 232 participants, 132 were deemed to have no risk for LS, 71 were identified with stage 1 LS, and 29 with stage 2 LS. Age, sex, weight, height, and BMI were not significantly different among the three groups (Table 1).

**Table 1:** Analysis of subject demographic characteristics in three groups according to locomotive syndrome risk

Characteristics	All	No risk	Stage 1	Stage 2	*P*- value
Number of subjects	232	132	71	29	
Age (year)	63.9	61.8	63.7	66.2	0.106
Sex (male/female)	101/131	62/70	36/35	11/28	0.682
Height (cm)	166.7 ± 8.58	167.5 ± 8.42	166.2 ± 8.74	166.5 ± 9.11	0.910
Weight (kg)	67.04 ± 11.92	67.8 ± 10.43	66.9 ± 13.72	66.73 ± 11.62	0.152
BMI (kg/m^2^)	24.3 ± 3.23	24.3 ± 3.47	24.2 ± 4.23	24.1 ± 3.61	0.068

Data are presented as mean ± SD unless otherwise specified. BMI indicates body mass index

LBP prevalence (%) showed a significant increase with higher LS risk stages, being 15.9% in the group with n risk, 32.1% in stage 1, and 44.8% in stage 2 (*P* = 0.0012). Increasing LS risk stages led to a significant rise in the VAS score (mm) for LBP, recorded at 7.45 ± 13.4 for no risk, 17.9 ± 18.3 for stage 1, and 25.45 ± 22.8 for stage 2 (*P* < 0.0001). While the prevalence of sciatica remained similar across the three groups (1.56% vs. 2.5% vs. 5.7%, *P* = 0.471), a significant increase in the VAS score (mm) for sciatica with advancing LS risk stages (3.24 ± 11.3 vs. 10.5 ± 17.6 vs. 21.2 ± 24.3, *P* < 0.0005).

With each stage of increasing LS risk, back muscle strength (kg) significantly decreased (82.4 ± 30.9 vs. 77.5 ± 26.3 vs. 65.1 ± 23.6, *P* = 0.0481). The data showed significantly increased in the 10-m gait time (s) (4.42 ± 0.51 vs. 4.91 ± 0.82 vs. 5.34 ± 0.98, *P* < 0.0002), and the 3-m TUG (s) followed the same trend (5.69 ± 0.74 vs. 6.41 ± 0.84 vs. 6.81 ± 0.92, *P* < 0.0002). Additionally, there was a tendency to decrease for the percentage (%) of maximum stride with higher LS risk stages (81.3 ± 4.12 vs. 73.3 ± 1.3 vs. 69.15 ± 7.63, *P* = 0.0001).

**Fig. 1:**
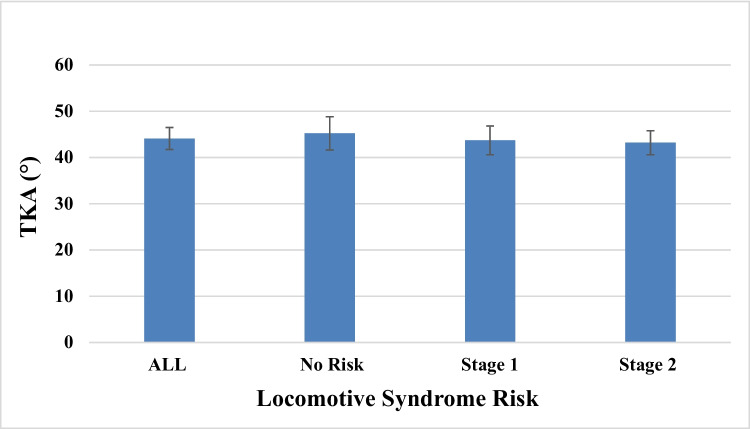
The association of locomotive syndrome risk with thoracic kyphosis angle (TKA)

**Fig. 2:**
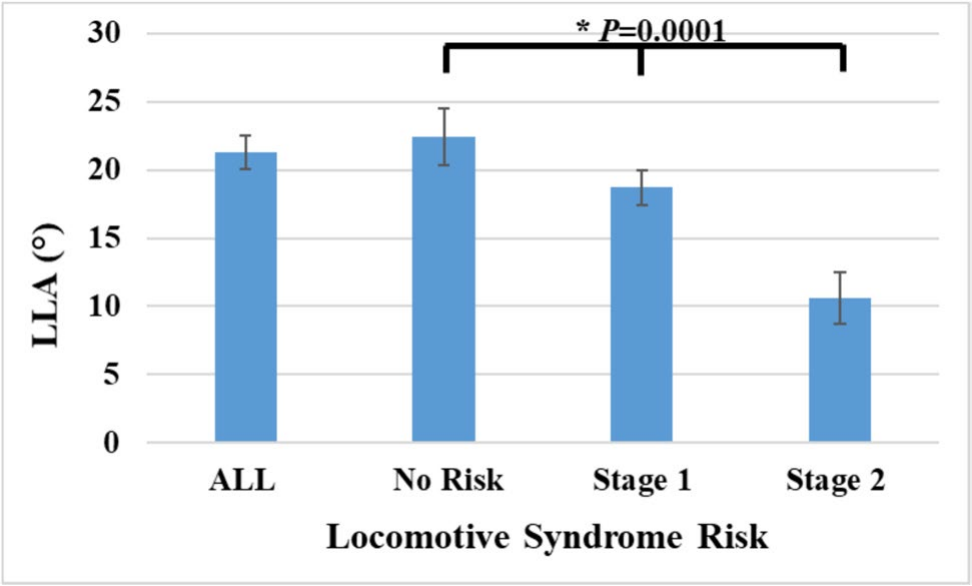
The association of locomotive syndrome risk with lumbar lordosis angle (LLA)

Radiographic measurements were taken for thoracic kyphosis and lumbar lordosis. Analysis indicated that TKA was consistent across the three groups (Fig. 1), but LLA decreased steadily with higher LS risk stages (*P* = 0.0001) (Fig. 2). The study showed that LDH decreased gradually and steadily as LS risk stage increased except for at levels L1–L2 and L5–S1 (Table 2)

**Table 2:** Comparative analysis of lumbar disc height at each level based on locomotive syndrome risk stage

Level (mm)	All	No risk	Stage 1	Stage 2	*P*- value
L1-L2	8.35 ± 2.32	8.47 ± 2.43	8.98 ± 1.9	8.91 ± 2.31	0.651
L2-L3	8.78 ± 2.4	9.32 ± 2.23	8.56 ± 2.12	8 ± 2.33	0.031*
L3-L4	8.92 ± 2.51	9.41 ± 2.61	8.53 ± 2.3	7.92 ± 2.46	0.0335*
L4-L5	8.67 ± 2.33	9.3 ± 2.44	8.66 ± 2.62	8.1 ± 2.53	0.001*
L5-S1	8.21 ± 3.21	8.8 ± 2.2	8.61 ± 1.82	8.56 ± 1.78	0.723

Data are presented as mean ± SD unless otherwise specified. ^∗^Statistically significant

**Fig. 3:**
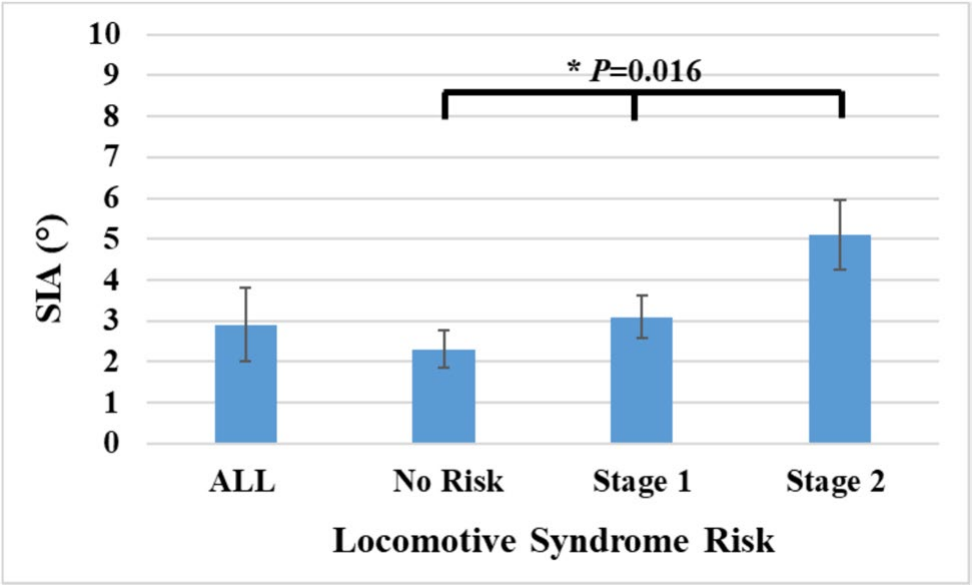
The association of locomotive syndrome risk with spinal inclination angle (SIA)

LOF prevalence significantly increased with higher LS risk stages across all segments (Table 3). Finally, the tendency of SIA was to increase with higher LS risk stages (*P* = 0.0167), peaking in those at stage 2 (Fig. 3)

**Table 3:** Prevalence rates of lumbar osteophyte formation in relation to locomotive syndrome risk stages

Osteophyte formation	All	No risk	Stage 1	Stage 2	*P*-value
Positive/negative	369/689	204/428	104/178	62/83	
Prevalence	35.1%	32.2%	37.1%	42.8%	0.038*

∗Statistically significant

## Discussion

LS is a growing concern for the elderly population, with significant implications for their health and quality of life. The interplay between aging, spine sagittal balance, and spinal degenerative changes underscores the complexity of this condition [16]. By implementing preventive measures, promoting regular physical activity, and ensuring early intervention for spinal issues, it is possible to reduce the impact of LS and support healthy aging. As the global population continues to age, addressing LS will remain a critical priority for healthcare providers, researchers, and policymakers.

In this study, we thoroughly examined the characteristics of each stage of LS risk to better understand the progression of this condition in the elderly population. The syndrome is categorized into three stages, each representing different levels of severity and risk for functional decline [3]. Understanding these stages is crucial for early detection, effective intervention, and prevention of further disability. This comprehensive look into the characteristics of each stage provides insights into how LS develops and progresses.

Results from the study illustrated a strong association between LS risk stages and spinal parameters, shedding light on how spinal health correlates with the progression of this condition among participants. This study is, to the best of our knowledge, the first to explore three groups (no risk, stage 1 LS, and stage 2 LS) in terms of the correlation between global spine sagittal balance and spinal degenerative changes in the Middle East and Arab world.

Participants diagnosed with LS exhibited notably severe LBP, reduced back muscle strength, and diminished physical ability. These symptoms not only impact daily functioning but also affect overall quality of life and independence. Weakness in the muscles supporting the spine can contribute to instability and difficulty in maintaining proper posture and movement [20, 21]. Based on the results, there was a substantial relationship observed between LS risk stage and increased spinal inclusion, lumbar lordosis, and progression of spinal degradation.

Following the onset of back muscle weakness, a notable characteristic of LS is the development of a kyphotic lumbar alignment, and LOF due to reduced lumbar disc height. In response to lumbar kyphotic changes, the body often makes compensatory adjustments, like reducing thoracic kyphosis. By decreasing the thoracic kyphosis, the body can counteract the increased forward tilt caused by lumbar kyphosis, helping to realign the spine’s overall sagittal profile [21]. This compensation helps individuals maintain their ability to bend forward and perform daily activities without excessive strain on the lumbar spine. If the compensatory adjustments fail to occur in response to lumbar kyphosis, individuals may experience significant biomechanical challenges that can impact their posture and overall spinal health [21]. According to Muramoto et al., the effect of spinal sagittal balance on LS and SIA was more significant among LS subjects [22], and Imagama et al. similarly concluded that maintaining proper spinal sagittal alignment, muscle strength, and faster 10-m gait speed can enhance body balance and lower the risk of falls [6]. Overall, studies indicate that progression in LS stage risk is associated with increased muscle weakness and SIA [13, 23], emphasizing kyphosis as a key factor influencing these aspects and highlighting the correlation between global spinal alignment and back muscle strength in the context of LS.

Back muscle weakness exacerbates the challenges posed by lumbar kyphosis, as the muscles are unable to adequately support the spine and maintain its normal alignment [24]. Weak muscles struggle to counteract the abnormal curvature, leading to further misalignment and increased stress on the lumbar discs. Over time, this added pressure can cause the discs to degenerate and lose height, further compromising spinal integrity and function. As a result, the space between vertebrae narrows, potentially compressing nerves and contributing to pain and reduced mobility [25]. The loss of disc height can also affect the stability and flexibility of the spine, further impairing an individual’s ability to perform daily activities comfortably [25]. Despite ongoing research, no previous reports have found a connection between global spine sagittal balance and the locomotor physical function of each LS risk stage in Middle East or Arab elderly populations.

There is an association between back muscle weakness and reduced LDH. As a result, performing exercises to strengthen the back muscles may help prevent LS in relatively healthy older individuals, ensuring the maintenance of lumbar lordosis and spinal alignment, and contributing to a higher quality of life. In this context, light strengthening exercises could be advantageous in maintaining lumbar lordosis, given that excessive back muscle strengthening exercises might LOF due to lumbar degeneration resulting from repeated heavy loading [26].

There were some limitations in this study, specifically, it had a small sample size and was conducted at a single site. Participants’ recruitment for this study proved challenging, particularly due to the time, patience, and effort required from participants, resulting in a small sample size. Furthermore, the study was conducted exclusively among individuals of the Arab race. Finally, despite the classification system encompassing three stages, our study was limited to analyzing the conditions of patients in the initial two stages due to the absence of stage 3 cases. Despite these limitations, the study’s results on the correlation between global spine sagittal balance, spinal degenerative changes, and LS risk are valuable for enhancing elderly population health management.

## Data Availability

Data are available from the corresponding author upon reasonable request.
